# Edges of human embryonic stem cell colonies display distinct mechanical properties and differentiation potential

**DOI:** 10.1038/srep14218

**Published:** 2015-09-22

**Authors:** Kathryn A. Rosowski, Aaron F. Mertz, Samuel Norcross, Eric R. Dufresne, Valerie Horsley

**Affiliations:** 1 Department of Molecular, Cellular and Developmental Biology, Yale University, New Haven, CT 06520, United States; 2 Department of Dermatology, Yale University, New Haven, CT 06520, United States; 3 Department of Physics, Yale University, New Haven, CT 06520, United States; 4 Departments of Mechanical Engineering and Materials Science, Chemical and Environmental Engineering, and Cell Biology, Yale University, New Haven, CT 06520, United States.

## Abstract

In order to understand the mechanisms that guide cell fate decisions during early human development, we closely examined the differentiation process in adherent colonies of human embryonic stem cells (hESCs). Live imaging of the differentiation process reveals that cells on the outer edge of the undifferentiated colony begin to differentiate first and remain on the perimeter of the colony to eventually form a band of differentiation. Strikingly, this band is of constant width in all colonies, independent of their size. Cells at the edge of undifferentiated colonies show distinct actin organization, greater myosin activity and stronger traction forces compared to cells in the interior of the colony. Increasing the number of cells at the edge of colonies by plating small colonies can increase differentiation efficiency. Our results suggest that human developmental decisions are influenced by cellular environments and can be dictated by colony geometry of hESCs.

Human embryonic stem cells (hESCs) provide an *in vitro* system to model the processes that control the earliest stages of cell fate specification during human development. Furthermore, due to their ability to differentiate into multiple cell types when subjected to the appropriate environmental cues, hESCs hold remarkable potential for regenerative medicine^1^. Thus, establishing the environmental factors that influence hESC differentiation will illuminate processes that impact human development and is fundamental to future clinical application of hESCs.

Several factors have been shown to impact the maintenance or differentiation of hESCs. Currently, hESCs can be maintained on Matrigel- or laminin-coated substrates, in conditioned media from mouse embryonic fibroblasts^2^ or in media supplemented with basic fibroblast growth factor (bFGF) and inhibitors of bone morphogenic protein 4 (BMP4)^3^. Addition of several other soluble chemical factors to three-dimensional aggregates or adherent monolayers of hESCs can recapitulate developmental signals found in the early embryo and induce formation of all three germ layers in culture^4^. However, most of these differentiation protocols are inefficient and do not generate homogenous populations of cells^5^,^6^. Besides chemical factors, it has previously been reported that mechanical properties of the ECM play a role in the differentiation of isolated stem cells^7^,^8^. Additionally, it has recently been shown that physical confinement of hESCs by restricting the growth of adherent colonies to patterned circles leads to simultaneous differentiation into all three germ layers, which reproduces their arrangement in development^9^. We anticipate that mechanical interactions of cells with each other and with the matrix likely play an important role in determining their fate.

In order to understand the potential role of cell-cell interactions on fate decisions in these early embryonic cells, we quantified the spatial organization of hESC differentiation. To this end, we examined colonies of hESCs treated with BMP4 during the first 3 days of differentiation. Surprisingly, after 3 days of BMP4 treatment, differentiated cells are localized to the edge of hESC colonies and form a band of consistent width independent of the size of the colony. Live tracking of these cells throughout the differentiation time-course revealed that the differentiated cells in the band originated from the edge of the undifferentiated colony, suggesting that the environment at the edge of an undifferentiated colony is distinct from that of the interior. Indeed, we find that cells at the edges of undifferentiated colonies experience a different mechanical niche than cells in the interior of the colony: cells at the edge have stronger mechanical interactions with the extracellular matrix, quantified by traction force microscopy. Furthermore, we show that differentiation efficiency is improved by increasing the percentage of primed cells at the colony edge, by plating smaller colonies. Together, these data provide evidence of a link between spatial organization of pluripotent cells and their differentiation potential.

## Results

### Differentiation of hESCs occurs at the edge of colonies

**Figure 1:**
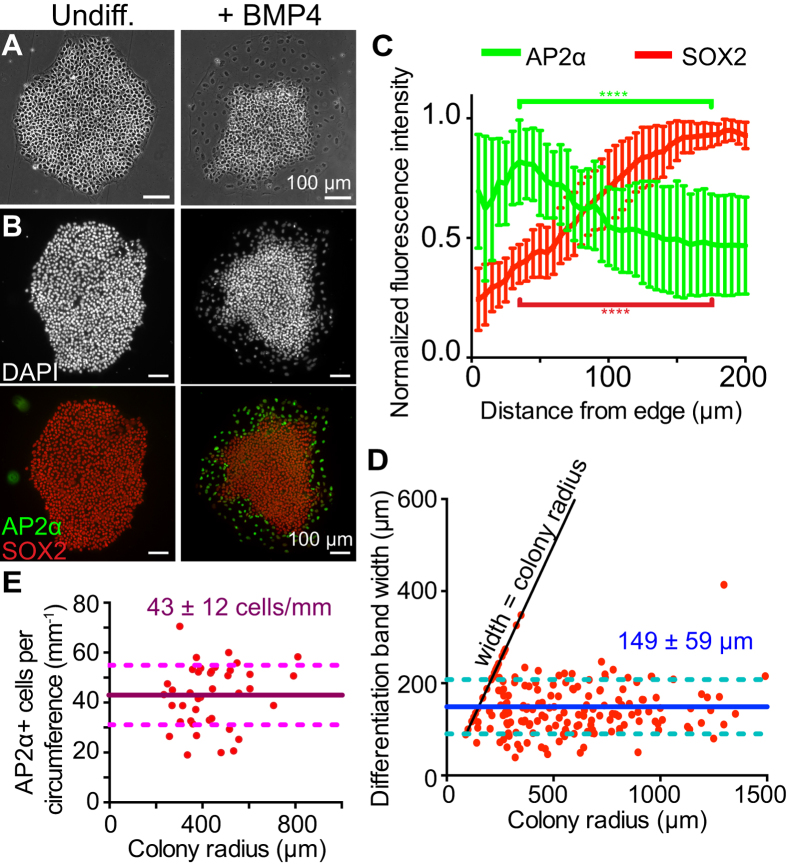
Differentiation occurs at the edge of hESC colonies. (**A**) Phase and (**B**) immunostaining images of hESC colonies when they are undifferentiated (top) and after 3 days BMP4 treatment (bottom). (**C**) Analysis of expression of pluripotency marker, SOX2, and differentiation marker, AP2α, after 3 days BMP4 treatment. Fluorescent intensity is plotted as a function of distance from the colony edge and normalized to the maximum intensity of each colony [*n *= 20 colonies, ****p < 0.0001 and represents statistics for AP2α (green) and SOX2 (red) levels between distance 35 μm and 175 μm from the edge using a two-tailed paired t-test]. Error bars represent S.D. from the mean. (**D**) The differentiation band width of hESC colonies plated on matrigel-coated glass coverslips or plastic dishes (red data points) reveals a constant average band width of differentiation of 149 μm (blue line) ± 59 μm (S.D.) (dotted cyan lines). The black line indicates a differentiation band width equal to the colony radius (*n *= 175 colonies). (**E**) The number of AP2α-positive cells quantified per circumference in a number of different colonies (red data points) shows a constant average number of cells per mm of circumference of 43 (maroon line) ± 12 cells (S.D.) (dotted magenta lines) (*n *= 11 colonies).

Previous reports have suggested that ectoderm differentiation occurs in response to several chemical stimuli including BMP4^10^. To examine ectoderm differentiation of hESCs in more detail, we treated H1 hESCs with BMP4 for 3 days. The cells at the edge of BMP4-treated hESC colonies displayed an expanded morphology, with larger nuclei and a greater cytoplasmic-to-nuclear ratio, compared to the densely packed cells within undifferentiated colonies^11^ and to those in the interior of BMP4-treated colonies (Fig. 1A). Immunostaining of colonies after BMP4 treatment with antibodies against several proteins expressed by pluripotent stem cells, including SOX2, OCT4, Nanog, and SSEA-3, revealed loss of expression of these pluripotent proteins in cells at the colony edge, while protein expression was maintained in cells localized to the colony interior (Fig. 1B; supplementary material Fig. S1A). In addition, the cells at the colony edge gained expression of the transcription factor AP2α (Fig. 1B; supplementary material Fig. S1B), which is expressed in ectoderm^12^. Quantification of fluorescence intensity, as a function of distance from the colony edge, in multiple colonies, confirmed that SOX2 intensity was consistently high in the center of the colony and dropped off at the edge as AP2α intensity increased (Fig. 1C).

To determine whether localized differentiation at the colony edge occurs when hESCs are induced to adopt other cell fates, we treated hESCs with Activin A to promote early endoderm^13^. Similar to BMP4 addition, Activin A treatment led to differentiation at the colony edge, as indicated by expression of the primitive streak transcription factor Brachyury (supplementary material Figs. S1A and S1B. Therefore, cells at the edge of the H1 hESC colonies appear to differentiate preferentially over those in the colony interior, irrespective of induced cell fate.

### Differentiation at the edge occurs within a band of constant width

To further characterize differentiation at the edge of BMP4-treated hESC colonies, we analyzed the width of differentiated cells in colonies of various sizes. As colonies grow and differentiate they form a rounded colony shape with a fairly consistent band around the colony edge. Surprisingly, we found that the average width of this band of differentiated cells was constant for colonies of various sizes (Fig. 1D, red data points), with an average width of 149 μm and a standard deviation of 59 μm (Fig. 1D, blue and cyan lines). The constant width of the differentiated cells at the edge suggested that the number of differentiated cells in each colony was proportional to colony circumference. Thus, we counted the number of AP2α-positive cells per colony and found that, when normalized to colony circumference, the number of differentiated cells was constant (Fig. 1E, red data points), averaging 42 cells per mm of circumference with a standard deviation of 12 cells (Fig. 1E, maroon and magenta lines).

**Figure 2:**
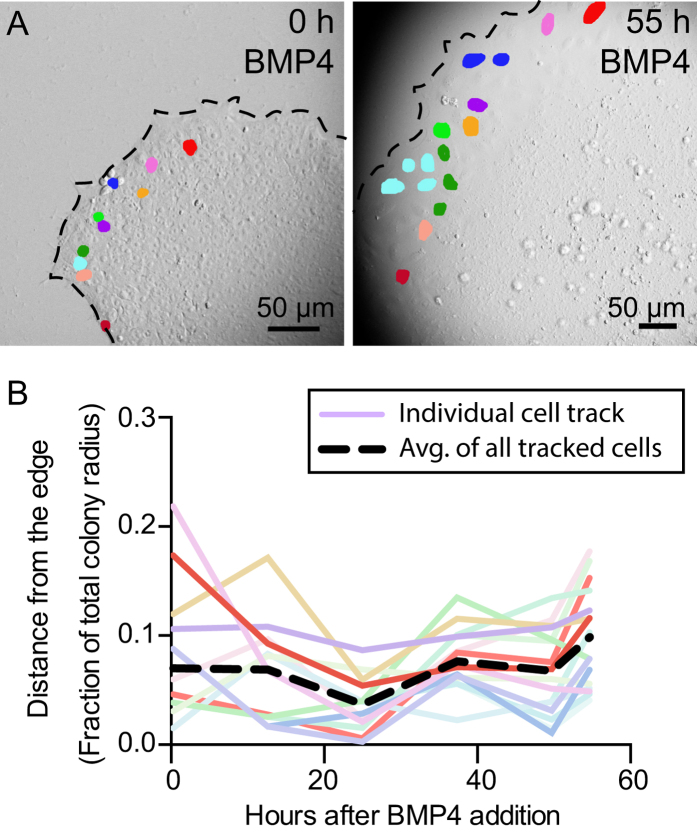
Differentiated cells of the hESC colony originate from the edge of the undifferentiated colony. (**A**) Single frames from live DIC imaging throughout the differentiation of an hESC colony. 16 cells were traced from the 55-hour time point to their original location at the 0-hour time point. The 16 cells are marked by the same colors in the top and bottom panels. Cells that divided are indicated as multiple dots of the same color. (**B**) Quantification of cell movement throughout the differentiation process, as represented by relative distance from the colony edge. The colors correspond to the cells indicated in (**A**).

To examine whether the band of differentiated cells in hESC colonies results from reorganization of cells within the colony post-differentiation, we tracked the location of individual cells in hESC colonies during BMP4-induced differentiation for 3 days using live cell imaging (supplementary material Movie M1). We used image analysis to mark individual cells within the differentiated band at the 60 hour time point. The location of the same cells was then followed through previous movie frames to the initial frame. (Fig. 2A). To account for cell movement due to colony growth from cell proliferation over time, we normalized the distance of the tracked cells from the colony edge to the total colony radius. Differentiated cells originated from a distance within the outer third of the undifferentiated colony edge (fraction of colony radius < 0.33) and remained within this third after differentiation (Fig. 2B). Thus, segregation of differentiated and undifferentiated cells does not occur through cell sorting but rather by edge cells differentiating and remaining at the colony edge.

### Cell-generated mechanical forces are predominantly at the edge of colonies

Our previous work analyzing mechanical interactions of cohesive keratinocyte colonies demonstrated the ability of E-cadherin-based cell-cell adhesions to localize traction forces to the colony periphery^14^. Like keratinocytes, hESCs colonies display characteristics of a polarized epithelium^15^ including E-cadherin-based cell-cell adhesions and integrin-based adhesions to ECM molecules^2^,^16^. Therefore, we sought to determine if cells at the edge of undifferentiated hESC colonies display distinct mechanical characteristics compared to cells in the colony interior.

**Figure 3:**
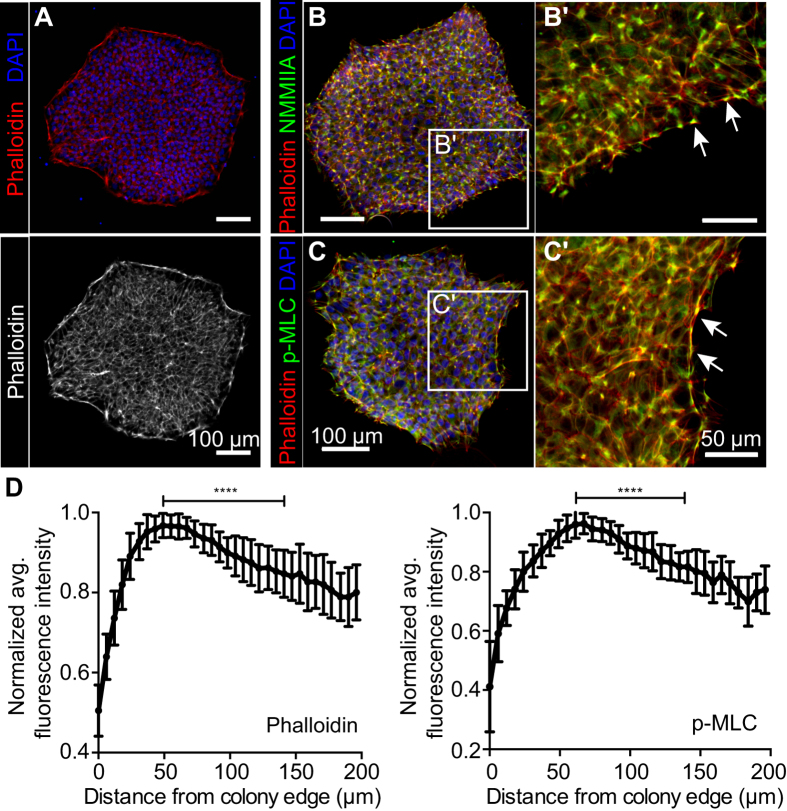
Cytoskeletal organization is distinct at edges of undifferentiated hESC colonies. (**A**) Phalloidin staining shows actin organization of a representative undifferentiated colony. (**B,C**) Non-muscle myosin IIA (NMMIIA) (**B**) and phospho-myosin light chain (p-MLC) (**C**) staining of an undifferentiated colony, localized to actin with phalloidin staining. White arrows indicate strong co-localization of myosin with actin. White boxes show where (**B′,C′**) are located in (**B–D**) Quantification of phalloidin and co-localized pMLC levels throughout the colony as a function of distance from the edge. (For phalloidin, *n *= 38 colonies, ****p < 0.0001 and represents statistics for levels between distance 49 μm and 141 μm from the edge using a Wilcoxon signed-rank test. For p-MLC, *n *= 20 colonies, ****p < 0.0001 and represents statistics for levels between distance 61 μm and 141 μm from the edge using a Wilcoxon signed-rank test). Error bars indicate S.D. of the mean.

Internal and external physical forces can act through the cytoskeleton to affect cell behavior^17^. In particular, actin filaments generate forces that drive changes in cell shape and mechanics through their interaction with myosin molecular motors. Thus, we stained undifferentiated colonies with fluorescently labeled phalloidin, which binds to F-actin, and immunostained with antibodies against non-muscle myosin IIA (NMMIIA) or phospho-myosin light chain (p-MLC), a mark of the activated form of myosin^18^. Interestingly, the organization of actin at the edge of the colony is distinct from that of the interior of the colony (Fig. 3A). Quantification of phalloidin expression within multiple colonies revealed a strong peak of F-actin near the edge (Fig. 3D). Elevated actin localization at the colony perimeter was accompanied by strong colocalization of NMMIIA and p-MLC at the edge (Fig. 3B,C). Quantification of the co-localization of activated myosin with actin, as a function of distance from the edge of the colony, also revealed a peak near the colony edge, further suggesting enriched actomyosin activity at the edge of undifferentiated hESC colonies (Fig. 3D).

**Figure 4:**
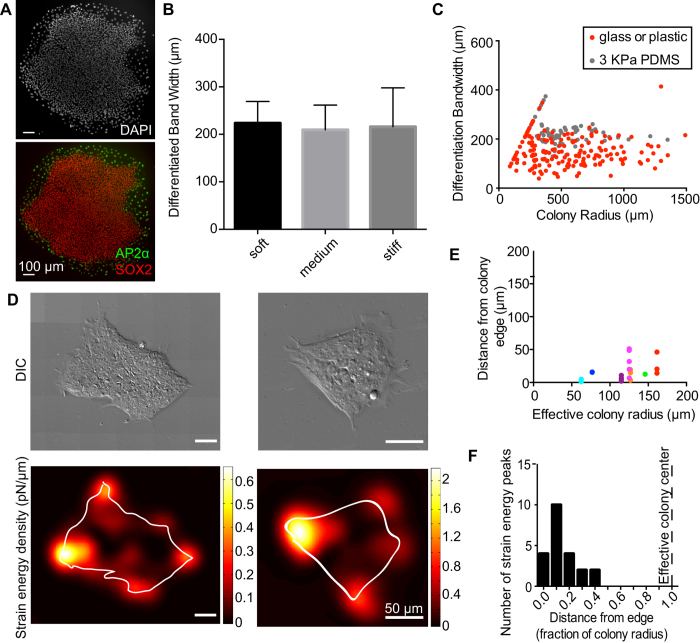
Cellular forces are predominantly on the edge of colonies. (**A**) Representative image of an hESC colony after 3 days of BMP4 treatment, grown on PDMS of 3 KPa stiffness. Nuclei are shown by DAPI staining and differentiation at the edge shown by immunostaining against AP2α and SOX2. (**B**) Bar graph of average differentiation band width on PDMS substrates of different stiffnesses. Soft is measured as 3 KPa, medium is estimated to be 30 KPa and stiff to be 100 KPa. (**C**) The differentiation band width of hESC colonies plated on matrigel- coated 3 KPa PDMS substrates (gray), overlaid on the data points from matrigel-coated glass coverslips or plastic dishes (red, as seen in Fig. 1D). (**D**) DIC images (top) and corresponding strain energy densities (bottom) of two undifferentiated hESC colonies, measured by traction force microscopy. 1 pN/μm = 10^−6^ J/m^2^. (**E**) Quantification of localization of peak regions of strain energy hESC colonies shows maximum strain energy peaks are near the edge of the colony, generally within 50 μm. (*n *= 7 colonies). (**F**) Histogram of distance from the edge, normalized by total colony effective radius, for peaks of strain energy within the colony. The dashed vertical line indicates the effective colony center. All measured strain energy maxima are closer to the edge than to this center.

To determine if the changes in actin and activated myosin at the colony edge translated into altered mechanical forces between the ECM and cells at the colony edge, we performed traction force microscopy (TFM) to measure forces applied to the substrate by cells in colonies of undifferentiated hESCs^19^. Since TFM is performed on PDMS substrates with low Young’s moduli, we analyzed hESC differentiation on matrigel-coated PDMS substrates. As with cells plated on glass and plastic substrates, differentiation of hESCs occurred at the colony edge on PDMS substrates (Fig. 4A). The differentiation band width was unaffected when cells were plated on PDMS substrates with different rigidities (3 KPa, 30 KPa, or 100 KPa) (Fig. 4B), which is consistent with other reports suggesting that cells are less responsive to substrate stiffness when plated on PDMS^20^,^21^. Although the average band width of differentiated hESCs on PDMS substrates was higher than we had previously measured on glass or plastic, the differentiation band width was within the range of the stiffer substrates (Figs 1D and 4C).

To measure traction stresses of undifferentiated hESC colonies, we plated hESCs on a Matrigel-coated elastic 3 KPa PDMS gels with embedded fluorescent beads^22^. Cell-ECM traction forces result in deformation of the substrate and concomitant displacement of the embedded beads relative to their “unstressed” positions after removing the cells with trypsin. Given the size of hESC colonies, this analysis required imaging and stitching up to 50 fields of view to fully analyze traction forces generated by hESC colonies. These images allowed us to calculate in-plane traction stresses from measured bead displacements and from the elastic properties of the substrate. Using the bead displacements and traction stresses, we calculated the strain energy density, the amount of work per unit area performed by the colony to deform the substrate (Fig. 4D). Our TFM measurements indicated that the majority of in-plane traction forces were localized primarily at the colony edge and pointed inward toward the center of the colony. To quantify localization of strain energy in multiple colonies, we located peaks of strain energy density and measured the distance of these points from the colony edge (Fig. 4E). In all colonies measured, the majority of regions of maximum strain energy were within 50 μm of the colony edge (Fig. 4E). When distance from the edge is normalized as a fraction of total colony radius, all colonies measured show strain energy peaks within the outer half of the colony (<0.5 of the colony radius) (Fig. 4F). These data are consistent with increased localization of cortical actin and activated myosin (Fig. 3), indicating that the mechanical state of the edge of undifferentiated hESC colonies is distinct from that of the interior.

### Plating cells in smaller colonies improves differentiation efficiency

**Figure 5:**
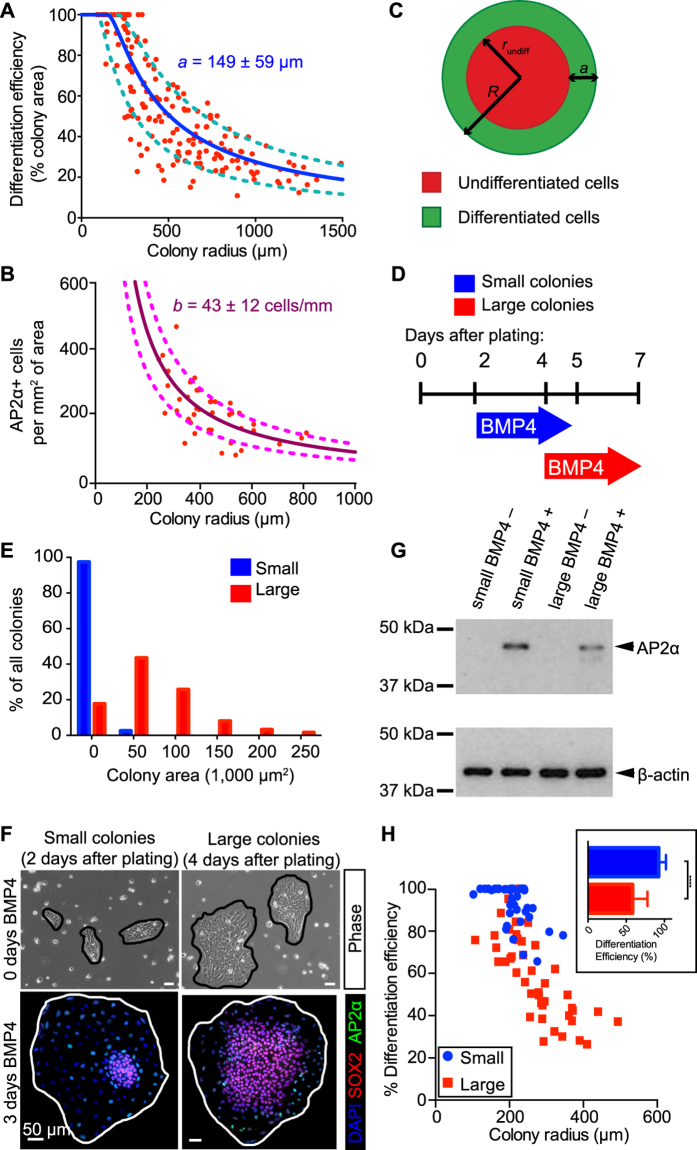
Plating hESCs in smaller colonies improves their differentiation efficiency. (**A**) Differentiation efficiency after 3 days BMP4 treatment, measured by percent of colony area that is undifferentiated (*n *= 175 colonies). Experimental data points (red) are plotted with theoretical curves assuming a constant differentiation band width of 149 μm (blue line) ± 59 μm (S.D.) (cyan lines). (**B**) Differentiation efficiency, measured by the number of AP2α^+^ cells per colony area (*n *= 11 colonies). Experimental data points (red) and plotted with theoretical curves assuming a constant number of AP2α^+^ cells per circumference of 43 cells (maroon line) ± 12 cells (S.D.) (magenta lines). (**C**) Schematic of idealized circular colony with radius, *R*, and differentiation band width, *a*. If *R* is close to *a*, then the radius of the undifferentiated cells in the middle, *r*_undiff_, will be small. (**D**) Scheme for controling colony size through single cells. (**E**) Quantification of average colony size shows colonies are smaller 2–3 days after plating than 4–5 days. (**F**) Phase (top) and immunostaining (bottom) images of representative small (left) and large (right) colonies. After 3 days of BMP4 treatment, smaller colonies have a greater percentage of differentiated cells. (**G**) Representative western analysis of AP2α levels with β-actin as loading control. Smaller colonies show greater expression of AP2α compared to larger colonies. Four independent experiments were performed. (**H**) Quantification of differentiation efficiency, measured by percent of colony area. Smaller colonies have greater efficiency and follow a curve similar to that in panels (**A**) and (**B**). Inset: average differentiation efficiency of small (red) versus large (blue) colonies. (*n *= 83 colonies, ****p < 0.0001 and represents statistics using a Mann-Whitney test).

Since differentiation of hESCs occurred predominantly at the edge of cohesive colonies, we hypothesized that smaller colonies—with a greater percentage of cells at the colony edge—would have a greater percentage of differentiated cells compared to larger colonies. We calculated the efficiency of differentiation as the percentage of the total colony area containing cells with a differentiated morphology. Colonies with radii smaller than 250 μm were completely differentiated, while ~20% of the area of large colonies with radii ~1,000 μm was comprised of differentiated cells (Fig. 5A, red data points). The differentiation efficiency transitioned smoothing between colonies with radii spanning these two extrema, indicating a relationship between colony radius and differentiation efficiency. Additionally, when we plotted differentiation efficiency as AP2α positive cells per colony area, we saw a similar downward trend as colony size increased (Fig. 5B, red data points).

Given the relationship between differentiation efficiency and colony size, we sought to determine if differentiation of hESCs could be described using a simple model. We modeled colonies as circles with total radius *R*, and the differentiated cells would generate a band of width *a* (Fig. 5C). In this case, geometry dictates that the differentiation efficiency could be described by the relationship: 
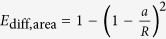
(1). Using this equation to calculate the differentiation efficiency using the measured band width of 149 ± 59 μm (Fig. 1D) and a range of *R* values well captures the downward trend of measured differentiation efficiencies in hESC colonies (Fig. 5A, blue and cyan lines). Furthermore, since the number of AP2α+ cells per colony circumference, *b*, is constant (Fig. 1E), we can estimate the number of AP2α+ cells using: 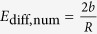 (2). Over a range of *R* values and using the measured AP2α+ cells per circumference value of 45 ± 8 cells/mm (Fig. 1E), the experimental data fall within this relationship (Fig. 5B, maroon and magenta lines). These data and analyses reveal that differentiation efficiency is strongly influenced by colony size and that despite the asymmetric nature of undifferentiated colonies, the geometry of colonies influences differentiation.

While restricting colony size can lead to loss of hESC pluripotency^23^, whether colony size can be manipulated to promote differentiation at the colony edge is unknown. Since colony area is inversely related to differentiation efficiency, we sought to determine if small hESC colonies, containing the majority of cells at the colony edge, have enhanced differentiation efficiency compared to a population of larger colonies. We plated hESCs as single cells and induced differentiation with BMP4 treatment after 2–3 (for small colonies) or 4–5 (for larger colonies) days in culture. After 3 days, nearly 100% of the colony radii were less than 130 μm, while after 5 days, colonies were larger, with the majority of radii ranging from 130–220 μm (Fig. 5D,E). Immunofluorescent analysis of SOX2 and AP2α expression in the smaller colonies after 3 days of differentiation revealed fewer SOX2+ cells and a greater number of AP2α+ cells at the edge of small colonies than in the larger colonies (Fig. 5F). Consistent with these immunofluorescence results, Western blot analysis of protein lysates from small colonies displayed elevated AP2α expression compared to larger colonies (Fig. 5G). Quantification of differentiation efficiency revealed that smaller colonies plated for 2 days clustered above 60% efficiency with the majority of colony area composed of 100% differentiated cells (Fig. 5G). By contrast, larger colonies displayed a wider range of both colony size and differentiation efficiency, ranging from 20% to close to 100% (Fig. 5H). Similar to the results in Fig. 1D, the differentiation efficiency of larger colonies correlated with colony size. Thus, differentiation of hESCs can be regulated by controlling the proportion of cells at the edge of colonies.

## Discussion

Our results show that edges of hESC colonies display a consistent spatial pattern of differentiation when soluble cues are provided. Furthermore, we find that the higher differentiation potential seen in cells at the edge of undifferentiated hESC colonies is accompanied by a distinct cortical actin organization, enhanced activation of myosin, and strong traction forces between the cells and ECM. These data suggest a unique mechanical nature of cells at the colony edge, which primes them for differentiation. By exploiting the character of the colony edge, we show that differentiation efficiency of hESCs follows a quantitative scaling to the amount of colony edge and can be improved by utilizing the nature of the colony edge. Unlike mechanical studies on single stem cells^16^,^24^, our data highlight the influence of both cell–ECM interactions and cell–cell interactions on the mechanical nature of cohesive hESC colonies and their importance in determining differentiation capacity of cells within a colony.

Our data provide further evidence that mechanotransduction pathways likely coordinate with chemical signals to influence differentiation. Consistent with our model, modulation of mechanotransduction through ECM stiffness or induction of mechanical strain has been shown to induce loss of pluripotency in mouse embryonic stem cells (mESCs)^25^,^26^,^27^,^28^. However, in hESCs, the impact of mechanical pathways is less clear. In the absence of chemicals to induce differentiation, spontaneous loss of differentiation is reduced by adding oscillating strain to hESCs^29^ or by plating cells on stiffer substrates^24^. However, it is unknown how applied mechanical forces interact with paracrine signaling and exogenous chemical factors. Interestingly, manipulation of the size and shape of hESC colonies through ECM patterning may control lineage fate specification^9^,^23^,^30^, though whether these bioengineering strategies alter mechanical properties of hESCs has not been established. Interestingly, we see that traction forces of undifferentiated hESCs are associated with changes in colony curvature, suggesting that asymmetric colony shape may influence the mechanical properties of the colony edge.

Our data are consistent with a pattern of traction force generated by tightly cohesive keratinocyte colonies, which require E-cadherin to organize and maintain colony traction forces^14^. E-cadherin signaling via β-catenin has been shown to be important in maintenance of hESC pluripotency in a myosin-dependent manner^16^. In fact, RhoA-GTPase/Rho-associated coiled-coil-containing kinase (ROCK)/myosin-II signaling can alter tension of the actin cytoskeleton and regulate survival of individual hESCs^31^,^32^,^33^. We find that actin-myosin co-localization is enhanced at the edge of adhesive colonies and that actomyosin is organized into coordinated bundles spanning multiple cells, indicating that mechanotransduction pathways may mediate the function of E-cadherin junctions in these cells. Our efforts to block myosin II activity or E-cadherin led to loss of colony adhesion and maintenance, as has been previously reported^16^, and thus we were unable to analyze consequences on differentiation due to altered adhesion in hESC colonies.

There are a number of possibilities for how mechanical environment could alter the pluripotent state of ESCs. It has been suggested that Smad signaling is not homogenous within colonies treated with BMP4^9^,^23^. Soluble factor availability may play a role in this heterogeneity, however it is also possible that mechanical forces at the colony edge may influence paracrine and endocrine signaling to establish a different chemical microenvironment at the edge of the colony. This idea is consistent with the ability of strain to support hESC pluripotency via induction of TGFβ signaling^34^. Mechanotransduction may also alter signaling directly through mechanisms such as activation of the transcription factors YAP/TAZ^35^,^36^. Interestingly, YAP/TAZ can bind to phosphorylated Smad proteins to control their movement to the nucleus and activation of downstream signaling^37^. Furthermore, hESCs show differential nuclear versus cytoplasmic expression of YAP based on the rigidity of their substrate^38^.

While manipulating mechanics of hESCs within colonies is experimentally challenging given the differential colony thickness and rapid cell-proliferation rate^29^, certain *in vivo* and *ex vivo* experiments have shed light on how mechanics influences pluripotent cells. Application of external forces to *Drosophila* embryos can induce expression of developmental transcription factor Twist, supporting the ability of mechanotransduction to influence differentiation of pluripotent cells^39^,^40^. Traction forces like those we measure in hESC colonies and cohesive keratinocyte colonies are suggestive of a surface tension^41^ similar to that described for developing embryos^42^. Developing embryos also exhibit cellular rearrangements that coordinate developmental signaling and require cell–cell and cell–matrix interactions that are coupled to actomyosin networks^43^,^44^. External mechanical forces generated *in utero* by intrapulmonary distensions/thoracic movements and by constraint of ECM molecules are necessary for lung or for bone development, respectively^45^,^46^,^47^,^48^. Recently, external mechanical forces from the uterine wall were suggested to play an important role in axis establishment in early post-implantation embryos^49^. Furthermore, advances in the ability to measure forces in living tissues are starting to reveal how mechanotransduction pathways act *in vivo*^50^,^51^. Our work suggests that taking advantage of these pathways in hESCs may improve differentiation efficiencies, a step toward understanding developmental processes and an essential advancement toward regenerative therapies.

## Methods

### Human embryonic stem cell culture and differentiation

H1 hES cells (Yale Stem Cell Core) were grown in mTeSR1 media (Stem Cell Technologies) as adherent colonies on cell culture dishes or glass coverslips coated with Matrigel (BD Biosciences). Cells were maintained at 37 °C, 5% CO_2_, and 5% O_2_ and passaged every 5–7 days, either as colonies (with Dispase, Stem Cell Technologies) or as single cells (with Accutase, Stem Cell Technologies). Cells split as single cells were pre-treated for 1 hour with ROCK inhibitor (Y-27632, EMD) and plated in the same inhibitor, at a final concentration of 10 μM. Media was changed to mTeSR1 the next day. Undifferentiated hESC colonies were plated on either cell culture dishes, glass coverslips, or PDMS substrates of CY 52-276-A and CY 52-276-B (Dow Corning Toray) or mixtures of Sylgard 184 (Dow Corning). To initiate ectoderm differentiation, 0.5 nM of human recombinant bone morphogenic protein 4 (BMP4) (R&D Systems) in mTesSR1 media was added to monolayers of hESCs, as confirmed by DAPI staining, and subsequently refreshed each day of differentiation. To induce endoderm differentiation, H1 hESC colonies were switched to media containing DMEM/F12 (Invitrogen), 1X L-glutamine (Invitrogen) and 100 ng/ml Activin A (R&D Systems) and refreshed daily for 2 days.

### Immunofluorescence

hESCs were fixed in 3.7% formaldehyde for 10 minutes at room temperature (RT). They were then incubated for >30 minutes in a gelatin block containing 1X PBS (Invitrogen), fish gelatin (Sigma), normal goat serum (NGS) (Jackson), normal donkey serum (NDS) (Jackson), 0.1% bovine serum albumin (BSA) (Sigma) and 0.25% Triton X. NGS was removed when staining with goat antibodies. Blocked cells were then incubated in primary antibodies diluted in gelatin block as follows: SOX2 (1:100, StemGent), AP2α (1:200, Developmental Studies Hybridoma Bank), non-muscle myosin IIA (1:1,000, Cell Signaling), phospho-myosin light chain (1:200, Cell Signaling), OCT4 (1:300, Millipore), SSEA-3 (1:200, Millipore), Nanog (1:600, Cell Signaling), Brachyury (1:300, R&D Systems). Primary antibodies were incubated for either 1 hour at RT or overnight at 4 °C. Alexa Fluor secondary antibodies (Invitrogen) were incubated at a dilution of 1:250 for 45 minutes at RT in the dark. Phalloidin conjugated to Alexa Fluor 594 was incubated for 20 minutes at RT (1:40 in gelatin block, Invitrogen). Finally cells were either incubated with DAPI (1:1,000 in PBS, Pierce) and mounted to slides with ProLong Gold (Invitrogen), or mounted in ProLong Gold with DAPI (Invitrogen).

### Modeling Differentiation Efficiency

Assuming a constant width of the differentiation band, *a*, the percentage of differentiation by area is:


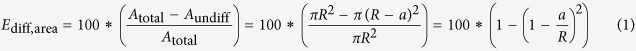


where *A* is area and *R* is the effective radius of the colony 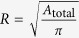.

Assuming a constant number of AP2α+ cells per unit of circumference, *b*, the calculation of differentiated cells per area is:


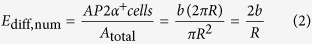


### Live imaging

Cells were imaged in a VivaView FL incubator fluorescence microscope, kept at 37 °C and 5% CO_2_. Differentiation media was changed daily. Cells were tracked throughout the frames by eye and their positions noted throughout the time course. We marked the edge of the colony, and the distance from the closest point on the edge to the center of the cell was used for the measurement of distance from the edge.

### TFM substrates

Round glass bottom dishes (Willco Wells) were coated with PDMS silicone gel: a 1:1 mixture of components CY 52-276-A and CY 52-276-B (Dow Corning Toray). After being degassed, the mixture was pipetted onto the dishes and spin-coated to the desired thickness of approximately 20 μm. The mixture was cured overnight at room temperature (RT) to obtain a gel with Young’s modulus 3 KPa^22^. To quantify deformation, the gel contained two layers of fluorescent beads (radius 100 nm, Invitrogen): one stationary layer between the glass and gel, and one layer around 20 μm above the dish, which moved in response to cellular forces. PDMS-coated dishes were soaked in 70% ethanol for 10 minutes and air-dried in a biosafety hood to sterilize before cell plating. Substrates were then coated with Matrigel, and cell culture was performed as described above.

### Traction force microscopy

Traction force microscopy was performed as described previously^41^. Briefly, hESCs and fluorescent beads embedded in the gel were imaged on a spinning-disk confocal microscope (Andor Revolution, mounted on a Nikon Ti Eclipse). Up to 50 DIC and fluorescent fields of view were stitched together to contain entire colonies of hESCs. The beads were tracked in MATLAB, and the gel displacement was calculated between the stressed (with cells attached) and unstressed states [after removing the cell with Trypsin (Invitrogen)]. From bead displacements and measured properties of the substrate, traction force magnitudes and directions, as well as strain energy densities, were calculated.

### Fluorescence intensity analysis

Fluorescent images were taken from stained cell slides on a Zeiss Epifluorescent Microscope at set exposures. These images were then processed by custom-written MATLAB code, which found the position of each nucleus and quantified total fluorescent intensity for each cell.

### Western blots

Protein was isolated from hESC samples through lysis with RIPA buffer. Protein was quantified with a BCA assay kit (Thermo), and equal amounts were loaded into an acrylamide gel. After running, samples were transferred to a nitrocellulose membrane and blocked in 5% milk for 1 hour at RT. The following primary antibodies were used overnight at 4°C: AP2α (1:50, Developmental Studies Hybridoma Bank), β-actin (1:2,000, Sigma). Anti-HRP secondaries were used at 1:5,000 (Jackson Labs) for 1 hour at RT.

### Statistical analysis

To determine significance between groups, comparisons were made using GraphPad Prism (GraphPad Software). D’Agostino and Pearson omnibus normality tests were performed to test for normal distribution. For normally distributed populations, a two-tailed paired t-test was used to compare values measured from the same cell colonies. For populations without normal distributions, the Mann Whitney test was used for unpaired results, and the Wilcoxon signed-rank test was used for paired results. *P *< 0.05 was accepted for statistical significance.

## Additional Information

**How to cite this article**: Rosowski, K. A. *et al.* Edges of human embryonic stem cell colonies display distinct mechanical properties and differentiation potential. *Sci. Rep.* **5**, 14218; doi: 10.1038/srep14218 (2015).

## Supplementary Material

Supplementary Movie 1

Supplementary Information
