# Supplementary material for: Edges of human embryonic stem cell colonies display distinct mechanical properties and differentiation potential
Source: Sci Rep. 2015 Sep 22;5:14218. doi: 10.1038/srep14218 (PMC4585749; doi:10.1038/srep14218)
Supplement: Supplementary Information [file srep14218-s2.pdf]

# Edges of human embryonic stem cell colonies display distinct mechanical properties and differentiation potential

Kathryn A. Rosowski[1], Aaron F. Mertz[3†], Samuel Norcross[1], Eric R. Dufresne[4, 3], and Valerie Horsley[1, 2*]

## Supplemental Information

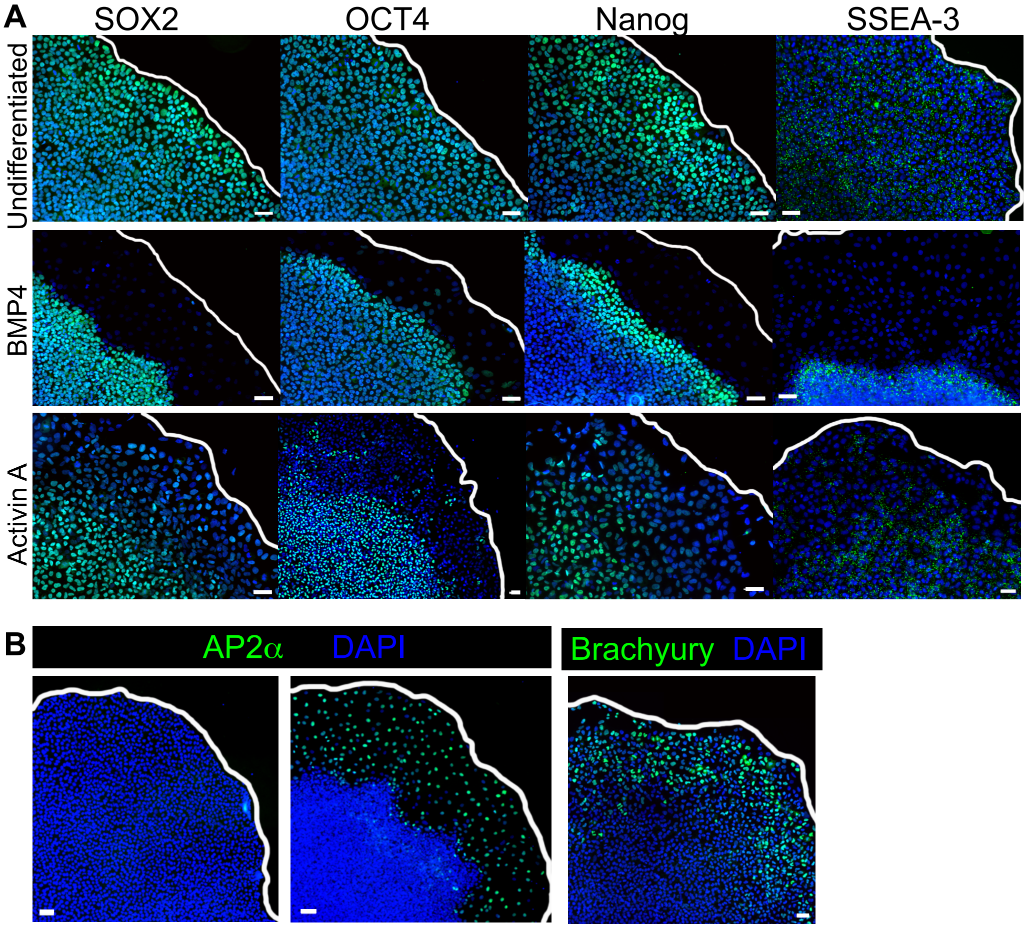


***Figure S1. Differentiation at the edge occurs after addition of multiple chemical factors***

A,B) Immunostained images of hESC colonies after 3 days of treatment with two differentiation cocktails: BMP4 for ectoderm differentiation and Activin A for endoderm differentiation. A) Various pluripotency markers are shown in green. B) Ectoderm differentiation marker AP2α, and mesendoderm differentiation marker Brachyury are shown in green. The blue channel shows DAPI staining. The white line indicates the edge of the colony. Scale bars represent 50 μm.

***Movie M1. Live imaging of hESC colonies throughout BMP4 differentiation***

DIC movie of a hESC colony as it differentiates. BMP4 was added just before the capture of the first frame. Time stamp shows progression of hours and minutes, through 15 minute frames. Scale bar represents 50 μm.
